# Vascular Access Device Infections: Current Management Practices and the Role of Multidisciplinary Teams at a Large Hospital in Northern Italy

**DOI:** 10.3390/antibiotics14010027

**Published:** 2025-01-03

**Authors:** Marta Colaneri, Lucia Galli, Martina Offer, Fabio Borgonovo, Giovanni Scaglione, Camilla Genovese, Rebecca Fattore, Monica Schiavini, Giovanni De Capitani, Maria Calloni, Arianna Bartoli, Antonio Gidaro, Chiara Cogliati, Spinello Antinori, Andrea Gori, Antonella Foschi

**Affiliations:** 1 Unit II, Department of Infectious Diseases, Luigi Sacco Hospital, ASST Fatebenefratelli Sacco, 20157 Milan, Italy; marta.colaneri@unimi.it (M.C.); galli.lucia@asst-fbf-sacco.it (L.G.); scaglione.giovanni@asst-fbf-sacco.it (G.S.); genovese.camilla@asst-fbf-sacco.it (C.G.); fattore.rebecca@asst-fbf-sacco.it (R.F.); schiavini.monica@asst-fbf-sacco.it (M.S.); andrea.gori@unimi.it (A.G.); foschi.antonella@asst-fbf-sacco.it (A.F.); 2 Department of Biomedical and Clinical Sciences, Università degli Studi di Milano, 20157 Milan, Italy; martina.offer@unimi.it (M.O.); chiara.cogliati@asst-fbf-sacco.it (C.C.); 3 Unit I, Department of Infectious Diseases, Luigi Sacco Hospital, ASST Fatebenefratelli Sacco, 20157 Milan, Italy; borgonovo.fabio@asst-fbf-sacco.it; 4 III Division of Infectious Diseases, Luigi Sacco Hospital, ASST Fatebenefratelli Sacco, 20157 Milan, Italy; decapitani.giovanni@asst-fbf-sacco.it; 5 Division of Internal Medicine, Luigi Sacco Hospital, University of Milan, 20157 Milan, Italy; maria.calloni@asst-fbf-sacco.it (M.C.); bartoli.arianna@asst-fbf-sacco.it (A.B.); gidaro.antonio@asst-fbf-sacco.it (A.G.)

**Keywords:** vascular access device (VAD), bloodstream infections, CRBSI, CABSI, multidisciplinary team, VAD infection management

## Abstract

**Introduction**: Vascular access device (VAD)-associated infections, including catheter-related (CRBSI) and catheter-associated bloodstream infections (CABSI), present significant challenges in patient care. While multidisciplinary VAD teams (VATs) are equipped with protocols for managing these infections, adherence to these guidelines in real-life practice is inconsistent. This study aims to evaluate the alignment between actual VAD infection management practices and VAT-recommended protocols. **Methods**: We conducted a retrospective, single-center study at Luigi Sacco Hospital (May 2021–October 2023) involving non-ICU adult patients with diagnosed CRBSI or CABSI. VAT experts independently reviewed infection management choices, which were divided into eight specific procedural options. These options included variations in VAD removal, timing of repositioning, and combinations of antimicrobial lock therapy and systemic therapy. Concordance between real-life practices and VAT recommendations was evaluated using Cohen’s kappa coefficient. **Results**: Of 2419 VAD placements, 146 (6%) developed infections (84 CABSI, 62 CRBSI). Clinicians removed VADs in 66.4% of cases compared to 62.3% per VAT recommendations, with moderate overall agreement (Cohen’s kappa = 0.58). Analysis of the eight management categories revealed moderate to low alignment (unweighted kappa = 0.44, weighted kappa = 0.30) between real-life practices and VAT guidance, with slightly improved concordance in CRBSI cases. **Conclusions**: Our findings underscore a discrepancy between real-life VAD infection management and VAT-recommended protocols, suggesting a need for clearer, more accessible guidelines and increased multidisciplinary collaboration. Enhanced VAT consultation and simplified protocol dissemination may improve consistency in infection management and ultimately lead to better patient outcomes.

## 1. Introduction

The placement of vascular access devices (VADs) is a growing practice among hospitalized patients [1,2,3]. These devices are employed in diverse clinical scenarios for purposes such as intravenous therapy or blood sampling, underscoring their significance in healthcare.

Most VADs are peripheral venous catheters, including short peripheral catheters, long peripheral catheters, and midlines. Only a small number of patients require centrally inserted central catheters (CICCs), femoral central catheters (FICCs), peripherally inserted central catheters (PICCs), or totally implantable devices such as ports and PICC ports. Given their extensive use in clinical practice, it is not difficult to imagine that many patients will require a VAD during their hospitalization. However, like all medical devices designed to improve patient care, VADs are not without complications, with infections being a particularly significant concern. Specifically, catheter-related bloodstream infection (CRBSI) and catheter-associated bloodstream infection (CABSI) represent a major cause of healthcare-associated infections [4]. These events are not only frequent in Europe, with rates reaching up to 3.86 per 1000 catheter-days [5], but also have serious consequences, significantly increasing hospital stay lengths, associated costs [6], and patient morbidity and mortality [7].

Timely and accurate diagnosis of CRBSI and CABSI is crucial to reduce complications, but guidelines are scarce and controversial [8]. Although CRBSI and CABSI diagnosis and management are complicated issues and only a few experts can optimally manage them, real-life conditions frequently let the responsibility fall on the clinicians caring for the patients across the hospital wards. They often mistakenly rely just on their judgment to make crucial decisions in the management of VAD complications, avoiding seeking sufficient help.

In recent years, many European hospitals have established VAD teams (VATs) composed of multidisciplinary specialists, such as infectious disease physicians, internists, anesthesiologists, and nurses, who focus on the placement, management, and care of vascular access devices [9]. These teams follow guidelines [1,10] and leverage extensive clinical experience. In addition to placing various VADs using an insertion bundle [11], VATs ensure ongoing maintenance and monitor for potential complications. Another key responsibility is education: VATs should educate healthcare staff, providing guidance on best practices for minimizing VADs’ infection risk [12]. Moreover, participating in broader hospital quality improvement initiatives and developing protocols and guidelines based on evidence-based practices to ensure the highest standards of care are essential tasks within the VAT’s scope [13].

Although these concepts may seem well established, VATs are not universally available across all healthcare facilities. Even in hospitals where they do exist, VATs are not always consulted for managing complex VAD-related complications. The approach to VAD management likely differs between dedicated VATs and general hospital clinicians, who typically oversee patient care on hospital wards, yet there is a lack of data to confirm or refute this potential disparity.

To investigate this significant and underexplored issue, we retrospectively assessed all VAD placements at a large university hospital in Northern Italy. We analyzed the appropriateness of infection management by comparing how infections were handled versus how the hospital’s VAT would have managed them.

This comparison highlights the differences between the choices made by clinicians operating independently and those guided by VAT protocols, reinforcing the value of a structured, multidisciplinary approach to reducing complications and enhancing patient care.

Our primary objective was to describe the clinical and microbiological characteristics of the CRBSI and CABSI episodes of the last two years in a large university hospital. Our secondary objective was to define the concordance between the management practices observed following the diagnosis of CRBSI or CABSI in the real-life clinical practice of a large university hospital and the management practices recommended by the VAT of the same hospital.

## 2. Results

### 2.1. Clinical and Microbiological Characteristics of CABSI and CRBSI Episodes

Regarding the primary outcome, out of 2419 VAD placements during the study period, 146 VADs (6%) were infected. Particularly, there were 84 (3.4%) CABSI and 62 (2.6%) CRBSI.

Since every patient might be hospitalized multiple times and could be subjected to multiple VADs, the total number of patients having CABSI or CRBSI was 135: 74 CABSI and 61 CRBSI.

The demographic and clinical characteristics of the patients with CRBSI and CABSI are reported in Table 1, while the microbiological characteristics of the VAD infections together with the types and sites of VAD placements are reported in Table 2.

The patients were mostly female (55.6%), and the median age was 60 years [IQR 49.67]. The median Charlson Comorbidity Index was 6 [IQR 5.8], and the most frequent comorbidities were immunosuppression (89.6%), hypertension (67.4%), neurological disease (42.2%), and cardiovascular disease (40%). Moreover, eight patients (5.9%) out of 135 had a previous catheter infection, particularly five CABSI and three CRBSI.

The median length of stay was 18 days [IQR 12.29], and the mortality rate was 27.9% due to CRBSI and 23% due to CABSI (Table 1).

Among the types of catheters investigated, PICC was the most represented (34.2%). Regarding the microbiology, the main pathogens that caused VAD infection were coagulase-negative staphylococci (52.1%) and *Enterococcus* spp. (20.5%) (Table 2).

### 2.2. Observed Real-Life Procedures and VAT Agreement

Firstly, we considered the procedures in the macro categories of “Catheter Removal” and “Non-Removal”.

Concerning the frequency of catheter removal decisions, among the 146 VAD infections, the catheter was removed in 97 cases (66.4%), while it was not removed in 49 (33.6%). Differently, the VAT opted for removal in 91 cases (62.3%) and non-removal in 55 cases (37.7%).

Cohen’s kappa was computed, resulting in a value of 0.58 (95% CI: 0.44–0.72) and indicating moderate agreement.

**Figure 1:**
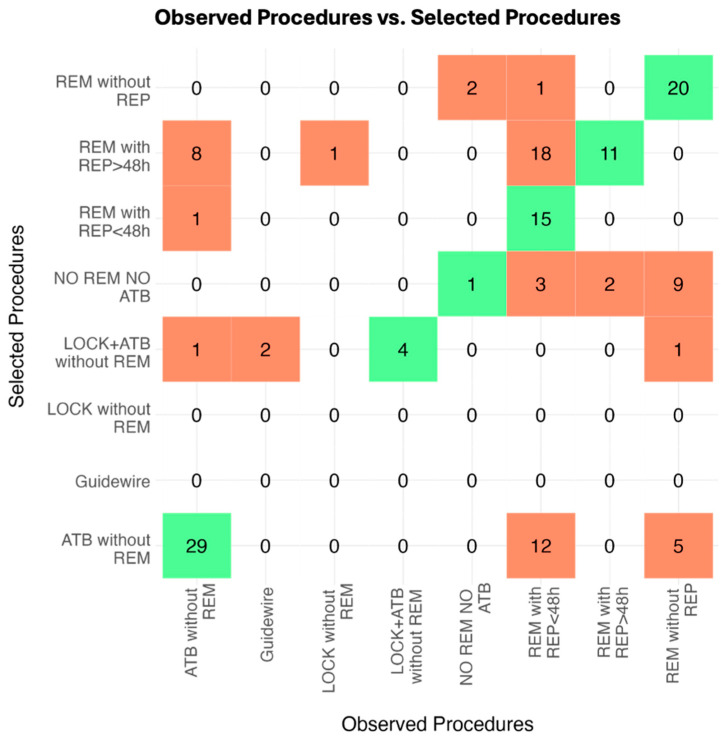
Confusion matrix: Observed procedures vs. the procedures selected by the VAT. A confusion matrix is a tool used to compare the 8 possible choices of management. It shows where those choices match (green squares) and where they do not (red squares). REM, catheter removal; REP, catheter repositioning; >48 h, at least 48 h after catheter removal and starting an effective antimicrobial therapy; <48 h, within 48 h of catheter removal and starting an effective antimicrobial therapy; Guidewire: catheter substitution over-guidewire; LOCK, antimicrobial lock therapy; ATB, systemic antimicrobial therapy.

Secondly, the eight observed procedures for the management of CABSI or CRBSI are reported in Table 3, while the procedures chosen by the VAT are described in Table 4. Figure 1 shows the confusion matrix, which provides a visual representation of the alignment or discrepancies between the real-life practices across hospital departments and the opinions developed by the VAT, highlighting areas of agreement and disagreement.

Cohen’s kappa analysis involved 146 VAD placements and assessed the VAT agreement on observed procedures in comparison to their selection methods. The unweighted kappa value of 0.44 (95% CI: 0.35–0.54) indicates moderate agreement (*p* < 0.001). The weighted kappa value of 0.30 (95% CI: 0.14–0.47) suggests a weaker agreement (*p* < 0.001).

Finally, Cohen’s kappa was then evaluated on patients with CABSI and CRBSI separately. For the CABSI group, positive unweighted and weighted kappa values were obtained, respectively 0.39 (95% CI: 0.28–0.51) and 0.22 (95% CI: 0.07–0.38) (*p* < 0.001). In patients with CRBSI, the weighted kappa value was 0.47 (95% CI: 0.34–0.60), while the unweighted was 0.37 (95% CI: 0.13–0.60) (*p* < 0.001), indicating higher agreement compared to the CABSI patients.

## 3. Discussion

Our exploration showed a rate of infectious complications regarding the inserted VADs of 6%, which is slightly higher than that observed by other studies, reporting pooled proportions of peripheral catheter-associated bloodstream infection under 1% [14,15] and under 3% for central venous catheters [16].

Regarding the microorganisms involved, we found that coagulase-negative staphylococci were the most prevalent pathogens; *Enterococcus* spp. was the second most frequent, followed by *Candida* spp. These findings are partially consistent with the existing literature, where coagulase-negative staphylococci frequently lead CRBSIs and CABSIs [17] due to their abundance on the skin and strong biofilm-forming abilities. However, *Staphylococcus aureus* is generally expected to be more prevalent in CRBSI cases, given its role as a common pathogen in device-related infections due to its virulence factors and association with more severe outcomes [18].

We found that 20.5% of CRBSIs and CABSIs were caused by *Enterococcus* spp., which is a higher proportion compared to other reports, although its incidence is increasing in recent years in the United States and Europe [19,20].

Finally, we observed that 27% of CRBSIs were caused by Candida spp. Although the incidence can vary depending on the center, other studies reported a lower rate of these infections, accounting for 11% of the CRBSIs [21,22].

Overall, we found a weak agreement between real-life management of CRBSI and CABSI across different wards of a large university hospital in Northern Italy and VAT opinion.

This significant result is not surprising. In fact, since VADs have become the most common procedure in hospitals across all the departments due to the increasingly aging population with poor venous capital, physicians frequently manage them under the misconception that it is an easy issue.

The reality is quite the opposite. These devices frequently encounter infectious complications to the extent that the term “epidemic” is not an exaggeration [23]. Unlike many other infectious syndromes, such as endocarditis, which is clearly within the realm of infectious disease specialists and requires strict diagnostic criteria at the bedside [24], VAD infections do not follow this pattern.

The diagnosis of CRBSI and even more of CABSI is particularly challenging and often controversial [25]. This is due to the lack of defined and undisputed diagnostic criteria, unlike other infectious diseases [26]. For CRBSIs, various guidelines exist [10,26,27,28], but there is no consensus among experts, with significant doubts remaining on key diagnostic points. For instance, some authors question the utility of DTP in defining CRBSI [8], suggesting that the type of infection and the implicated pathogen might influence the diagnosis differently [29,30,31].

As a result, the management of these infections is difficult to standardize, leading to confusion. For this reason, a common strategy is to remove the presumed infected VAD, but these removals are often inappropriate.

Our findings emphasize the necessity for a unified approach and shared understanding between the ward where the patient is treated and the VAT, which should be consulted not only for the placement of difficult VADs, urgently needed for life-saving therapies and blood draws, but also for actively discussing their management. Multidisciplinary discussion should become the cornerstone of VAD management because a single viewpoint often leads to flawed management, especially when guidelines are not unified and personal management approaches are based on individual beliefs.

Furthermore, our results highlight the need for simpler, more user-friendly internal guidelines. Clinicians cannot be expected to memorize and keep at hand the most recent, lengthy, and complex guidelines. Along with the multidisciplinary VAT service, there should be simple and coherent guidelines available for use until a collegial discussion can take place. We believe it is essential to draft internal guidelines that consider various real-life scenarios and are accessible to everyone. Including flowcharts and user-friendly diagrams could be particularly helpful in emergency situations across all wards. This might also alleviate some of the emotional stress and fear associated with managing VAD-related infections.

We acknowledge some limitations. Firstly, the retrospective nature of the design and the involvement of only three VAT experts may impact the generalizability of our findings, even if we consider that three experts, each from different clinical backgrounds, provide a sufficiently diverse perspective to understand the various approaches to the conditions under study.

Secondly, the diagnoses of CRBSI and CABSI were derived from clinical record reviews, which might have led to some misinterpretations of clinical situations that could have been differently assessed during real-time VAT consultations. However, we also note that VAT consultations are often based on electronic medical record reports, not just oral communication with the treating clinician, making our case review process quite reflective of real-life practice.

Additionally, the eight potential management options for CRBSI and CABSI might seem reductive compared to the myriad nuances encountered in daily clinical practice. Nevertheless, we believe that our simplification adequately covers many realistic possibilities. In conclusion, our study highlights the complexities and inconsistencies in the diagnosis and management of CRBSI and CABSI among VAT experts. Despite the limitations, the findings underscore the need for improved guidelines and multidisciplinary collaboration to enhance patient care. Moving forward, establishing clear, user-friendly protocols and fostering better communication between clinicians and VATs will be crucial in addressing these challenges.

## 4. Materials and Methods

### 4.1. Study Design and Clinical Setting

This retrospective, monocentric observational study took place at Luigi Sacco Hospital between 1 May 2021 and 1 October 2023. This study started after the establishment of a VAT in 2018, which implemented standardized procedures and data reporting formats. All devices were inserted according to the “Safe insertion of PICCs (SIP)” protocol [11].

### 4.2. Study Population

This study involved a review of medical records from non-ICU adult patients who were hospitalized and received various central venous access devices (CVADs), including PICC, FICC, and CICC, as well as peripheral venous access devices (PVADs), like long peripheral catheters and midline placements. Adults over the age of 18 years who had undergone CVAD or PVAD placement were included. Exclusion criteria were applied to patients who only received short peripheral cannulas or were admitted to the ICU.

The patients who were considered in our study were selected from a systematic registry of all VADs placed by VAT in clinical wards (infectious diseases, oncology, internal medicine, nephrology, etc.) and surgical departments (orthopedic surgery, cardiac surgery, general surgery, etc.). From this database, we selected only the patients who had episodes of CRBSI or CABSI during their hospitalization.

### 4.3. Ethics

This study was conducted in accordance with the Declaration of Helsinki and approved by the Luigi Sacco Hospital Institutional Review Board (Research Ethics Committee approval number 2021/ST/180).

### 4.4. Definitions

The definitions of CABSI and CLABSI used in this study were aligned with the 2024 Infusion Nursing Society Standards of Practice and the CDC’s National Healthcare Safety Network [32]. CABSI refers to BSIs associated with both PVADs and CVADs, excluding infections originating from other sites. These definitions serve as indicators of primary BSI in patients with a VAD placed either on the day of infection onset or the preceding day and maintained for more than two days.

CRBSI was identified using two criteria [1]: (i) the differential time to positivity—identical microorganisms are found in blood cultures drawn simultaneously from a peripheral vein and the VAD, with VAD cultures turning positive at least two hours before peripheral vein cultures; and/or (ii) the same microorganism is isolated from both the catheter tip and peripheral vein blood cultures.

Immunosuppression was defined as the presence of at least one among the following conditions: HIV, infection, hematopoietic stem cell transplantation (HSCT), solid organ transplantation (SOT), and autoimmune conditions.

The infections related to VAD placements could be more than the patients diagnosed with CRBSI and CABSI, since every patient could be hospitalized multiple times and could be, thus, subjected to multiple VAD placements.

### 4.5. Available Data

Electronic patient records provided demographic data (age, sex), clinical information (medical history, underlying conditions, treatments, outcomes), and laboratory findings. The laboratory results included biochemical markers (e.g., albumin levels) and microbiological data, such as SARS-CoV-2 tests, cultures, urine cultures, and colonization surveillance swabs. The details of VAD placements were recorded on standardized data sheets, documenting catheter type, number of lumens, insertion site, placement and removal dates, intra-procedural complications, incidences of CABSI and CRBSI, as well as reasons for catheter removal.

### 4.6. Study Procedure

Three experts from the VAT at Luigi Sacco Hospital, namely a physician specialized in infectious diseases, a nurse, and a physician specialized in internal medicine who were all core members of the VAT, met weekly for three months to actively discuss episodes of CABSI and CRBSI that occurred in the hospital over recent years (between 1 May 2021 and 1 October 2023). The three specialists, each independently, reviewed the infection management practices used in the hospital departments where patients with CRBSI and CABSI had been admitted. The three experts reviewing the cases were unaware of the patients’ outcomes; they were informed only after providing their opinions.

After reviewing the clinical details and patient histories from the electronic medical records, the three specialists shared their individual assessments and reached a consensus on a standardized procedure for handling cases of CRBSI or CABSI.

Specifically, they evaluated the clinical decisions made by their colleagues who had been responsible for the care of the patients and offered their expert opinion on each case’s management: they could either confirm their agreement with the actions taken or disagree. In this latter case, if they disagreed with the observed practice, they were subsequently asked what practice they would have implemented instead.

### 4.7. Potential Choices

A series of procedures that could have been carried out at the time of CABSI or CRBSI diagnosis were selected.

Firstly, the VAD could either be left in place or removed. This initial major division represents a significant milestone in the clinical decisions made regarding VAD management.

Secondly, there were more specific choices that could be implemented in real-life scenarios, which were provided as options to the experts. In particular, (i) the VAD could be removed and not repositioned; (ii) removed and repositioned after waiting an equal or greater time than 48 h; (iii) removed and repositioned within 48 h; (iv) repositioned over a guidewire; (v) not be removed, and antimicrobial lock therapy combined with systemic antibiotic or antifungal therapy could be applied; (vi) not be removed, and antimicrobial lock therapy could be applied without systemic antibiotic/antifungal therapy; (vii) not be removed, and systemic antimicrobial therapy could be applied; or (viii) not be removed, and no systemic therapy or lock therapy could be applied.

### 4.8. Statistics

Continuous variables are described as medians and interquartile range (IQR), while categorical variables are described as counts and percentages. Differences between infection groups (CABSI and CRBSI) are assessed using Mann–Whitney test and Fisher’s exact test, as appropriate. Statistical significance is accepted at the 5% level.

Descriptive tables were constructed based on the total number of VAD replacements and the total number of patients. For patient-level aggregation, categorical variables for each patient were grouped based on the presence of at least one positive observation across their replacements. Continuous variables were aggregated using the mean of the patient’s row values.

Agreement was evaluated as the concordance of the VAT opinion with the procedures observed in the population using unweighted Cohen’s kappa coefficient [33]. This assessment was conducted to evaluate agreement based on the presence or absence of concordance.

Further agreement analysis was conducted, focusing on the macro categories of ’Catheter Removal’ (VAD could be removed and not repositioned, removed and repositioned after waiting an equal or greater time than 48 h, and removed and repositioned within 48 h) and ’Non- Removal’ (VAD could be repositioned over a guidewire, not be removed, and lock therapy combined with systemic antibiotic therapy could be applied; not be removed, and antibiotic lock therapy could be applied; not be removed, and systemic antibiotic therapy could be applied; not be removed, and no systemic therapy or lock therapy could be applied).

Data were analyzed using R software, version 4.3.3.

## 5. Conclusions

In conclusion, our study reveals a weak alignment between the real-life management of CRBSI and CABSI across hospital wards and the approaches recommended by VAT specialists, underscoring the need for improved clinical guidelines and multidisciplinary collaboration. Simplified, accessible protocols alongside VAT consultation could significantly enhance the consistency and quality of care for VAD-related infections. Despite certain limitations, our findings highlight critical areas for intervention, supporting the development of streamlined guidelines and collaborative frameworks to improve patient outcomes.

## Figures and Tables

**Table 1:** Demographic and clinical characteristics of all the patients with a diagnosis of CABSI and CRBSI.

	Total (N = 135)	CABSI (N = 74)	CRBSI (N = 61)	*p* Value *
Age (IQR)	60 (49, 67)	62 (53, 67)	57 (46, 66)	0.129
Female (%)	75 (55.6%)	41 (55.4%)	34 (55.7%)	1.000
CCI (IQR)	6 (5, 8)	6 (5, 8)	7 (5, 8)	0.214
Comorbidity count (IQR)	4 (3, 5)	4 (3, 5)	4 (3, 5)	0.641
Cancer (%)	27 (20.0%)	8 (10.8%)	19 (31.1%)	0.005
Hematologic malignacies (%)	10 (7.4%)	8 (10.8%)	2 (3.3%)	0.113
Diabetes (%)	34 (25.2%)	16 (21.6%)	18 (29.5%)	0.324
CVD (%)	54 (40.0%)	27 (36.5%)	27 (44.3%)	0.382
COPD (%)	29 (21.5%)	15 (20.3%)	14 (23.0%)	0.834
Neurological disease (%)	57 (42.2%)	29 (39.2%)	28 (45.9%)	0.486
Mental disorder (%)	15 (11.1%)	6 (8.1%)	9 (14.8%)	0.275
Cirrhosis (%)	7 (5.2%)	4 (5.4%)	3 (4.9%)	1.000
CKD (%)	13 (9.6%)	8 (10.8%)	5 (8.2%)	0.772
Hypertension (%)	91 (67.4%)	54 (73.0%)	37 (60.7%)	0.143
Obesity (%)	15 (11.1%)	13 (17.6%)	2 (3.3%)	0.011
Immunosuppression (%)	121 (89.6%)	72 (97.3%)	49 (80.3%)	0.001
Previous CABSI/CRBSI (%)	8 (5.9%)	5 (6.8%)	3 (4.9%)	0.729
Albumin g/L (IQR)	16 (13, 21)	16 (13, 20)	17 (13, 22)	0.442
Missing	8	5	3	
LOS (day) (IQR)	18 (12, 29)	16 (12, 28)	21 (12, 29)	0.183
Missing	25	14	11	
Transferred from ICU (%)	8 (5.9%)	3 (4.1%)	5 (8.2%)	0.467
Death (%)	34 (25.2%)	17 (23.0%)	17 (27.9%)	0.554
Transferred to ICU (%)	7 (5.2%)	4 (5.4%)	3 (4.9%)	1.000

CCI, Charlson Comorbidity Index; CVD, cardiovascular disease; COPD, chronic obstructive disease; CKD, chronic kidney disease; CABSI, catheter-associated bloodstream infection; CRBSI, catheter-related bloodstream infection; LOS, length of stay; ICU, intensive care unit. * *p* value for numeric variables calculated using the Mann–Whitney test. *p* value for categorical variables calculated using the Fisher’s exact test.

**Table 2:** Microbiological characteristics of all the positioned VAD infections, type of VADs, and observed procedures.

	Total (N = 146)	CABSI (N = 84)	CRBSI (N = 62)	*p* Value *
Isolated pathogens				
*Staphylococcus aureus*	6 (4.1%)	1 (1.2%)	5 (8.1%)	0.083
*CoNS*	76 (52.1%)	47 (56.0%)	29 (46.8%)	0.316
*Streptococcus* spp.	4 (2.7%)	3 (3.6%)	1 (1.6%)	0.637
*Enterococcus* spp.	30 (20.5%)	16 (19.0%)	14 (22.6%)	0.680
*Enterobacteriaceae* spp.	17 (11.6%)	13 (15.5%)	4 (6.5%)	0.120
*Pseudomonas aeruginosa*	2 (1.4%)	0 (0.0%)	2 (3.2%)	0.179
*Bacillus* spp.	3 (2.1%)	2 (2.4%)	1 (1.6%)	1.000
*Candida* spp.	18 (12.3%)	1 (1.2%)	17 (27.4%)	<0.001
*Polymicrobial*	19 (13.0%)	8 (9.5%)	11 (17.7%)	0.213
VAD type				<0.001
Mid-thigh	45 (30.8%)	34 (40.5%)	11 (17.7%)	
Midline	36 (24.7%)	25 (29.8%)	11 (17.7%)	
Other CVADs	15 (10.3%)	8 (9.5%)	7 (11.3%)	
PICC	50 (34.2%)	17 (20.2%)	33 (53.2%)	

CoNS, coagulase-negative staphylococci; VAD, vascular access device. CVADs, central venous access devices; PICC, peripherally inserted central catheter. * *p* value for numeric variables calculated using the Mann–Whitney test. *p* value for categorical variables calculated using the Fisher’s exact test.

**Table 3:** Selected procedures—CABSI vs. CRBSI.

	Total (N = 146)	CABSI (N = 84)	CRBSI (N = 62)	*p* Value *
REM without REP	35 (24.0%)	22 (26.2%)	13 (21.0%)	0.558
REM with REP > 48 h	13 (8.9%)	5 (6.0%)	8 (12.9%)	0.156
REM with REP < 48 h	49 (33.6%)	25 (29.8%)	24 (38.7%)	0.290
Guidewire	2 (1.4%)	2 (2.4%)	0 (0.0%)	0.508
LOCK without REM	1 (0.7%)	0 (0.0%)	1 (1.6%)	0.425
LOCK + ATB without REM	4 (2.7%)	0 (0.0%)	4 (6.5%)	0.031
ATB without REM	39 (26.7%)	29 (34.5%)	10 (16.1%)	0.014
NO REM and NO ATB	3 (2.1%)	1 (1.2%)	2 (3.2%)	0.575

* *p* value for categorical variables calculated using the Fisher’s exact test. REM, catheter removal; REP, catheter repositioning; >48 h, at least 48 h after catheter removal and starting an effective antimicrobial therapy; <48 h, within 48 h of catheter removal and starting an effective antimicrobial therapy; Guidewire: catheter substitution over-guidewire; LOCK, antimicrobial lock therapy; ATB, systemic antimicrobial therapy.

**Table 4:** Procedures chosen by the VAT—CABSI vs. CRBSI.

	Total (N = 146)	CABSI (N = 84)	CRBSI (N = 62)	*p* Value *
REM without REP	23 (15.8%)	7 (8.3%)	16 (25.8%)	0.006
REM with REP > 48 h	38 (26.0%)	6 (7.1%)	32 (51.6%)	<0.001
REM with REP < 48 h	16 (11.0%)	8 (9.5%)	8 (12.9%)	0.596
Guidewire	0 (0.0%)	0 (0.0%)	0 (0.0%)	
LOCK without REM	0 (0.0%)	0 (0.0%)	0 (0.0%)	
LOCK + ATB without REM	8 (5.5%)	4 (4.8%)	4 (6.5%)	0.723
ATB without REM	46 (31.5%)	44 (52.4%)	2 (3.2%)	<0.001
NO REM and NO ATB	15 (10.3%)	15 (17.9%)	0 (0.0%)	<0.001

* *p* value for categorical variables calculated using the Fisher’s exact test. REM, catheter removal; REP, catheter repositioning; >48 h, at least 48 h after catheter removal and starting an effective antimicrobial therapy; <48 h, within 48 h of catheter removal and starting an effective antimicrobial therapy; Guidewire: catheter substitution over-guidewire; LOCK, antimicrobial lock therapy; ATB, systemic antimicrobial therapy.

## Data Availability

The data presented in this study are available by the corresponding author on request, provided that data privacy of the patients is preserved.
